# The Gut Microbiota and Immune System Relationship in Human Graft-versus-Host Disease

**DOI:** 10.4084/MJHID.2016.025

**Published:** 2016-05-01

**Authors:** Lucrezia Laterza, Gianenrico Rizzatti, Eleonora Gaetani, Patrizia Chiusolo, Antonio Gasbarrini

**Affiliations:** 1 Fondazione Policlinico A. Gemelli, UOC of Internal Medicine, Gastroenterology and Liver Diseases. L.go Gemelli, 8 Rome, Italy; 2 Fondazione Policlinico A. Gemelli, Institute of Haematology, L.go Gemelli, 8 Rome, Italy

## Abstract

Gut microbiota has gained increasing interest in the pathogenesis of immune-related diseases. In this context, graft-versus-host disease is a condition characterized by an immune response which frequently complicates and limits the outcomes of hematopoietic stem cell transplantations. Past studies, carried mostly in animals, already supported a relationship between gut microbiota and graft-versus-host disease. However, the possible mechanisms underlying this connection remain elusory. Moreover, strategies to prevent graft-versus-host disease are of great interest as well as the potential role of gut microbiota modulation. We reviewed the role of gut microbiota in the development of immune system and its involvement in the graft-versus-host disease, focusing on data available on humans.

## Introduction

Microbiota is the complex system of bacteria, archaea, viruses and fungi living in several body niches, such as skin, vagina, nose and mouth. However, the majority of microorganisms live in the digestive tract. Gut microbiota should be considered a real organ, accounting 100 times more genes than the host and being responsible for multiple functions and in particular of the metabolic and immune homeostasis.^1^

**Figure 1:**
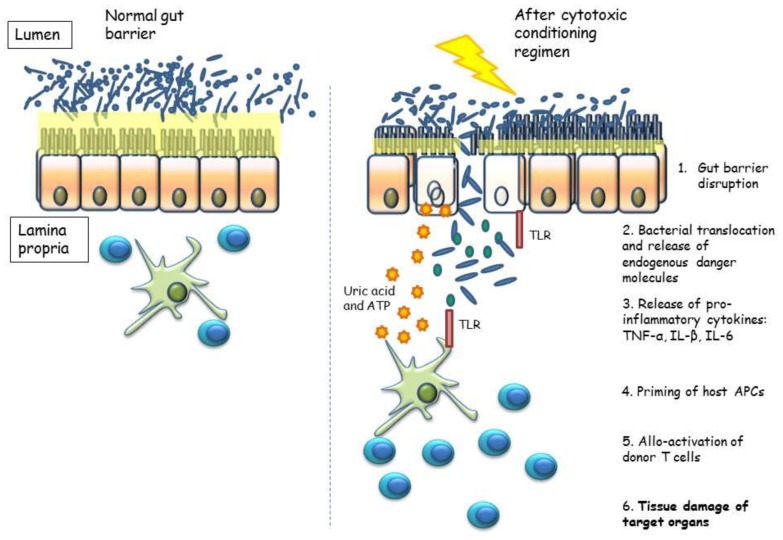
The gut barrier and its alterations during the pathogenesis of GVHD. The healthy gut barrier is essential to maintain the immune homeostasis. Total body irradiation and/or chemotherapy, used as conditioning regimen, lead to gut barrier disruption, damaging the mucus layer and the epithelium. Thus, bacteria and bacterial products such as lipopolysaccharide translocate in the lamina propria where, together with endogenous danger molecules released from damaged epithelial cells, activate host and/or donor antigen-presenting cells (APCs) which prime alloreactive donor-derived T cells, triggering the damage to target organs. Modified from Heidegger.^59^

Recent studies demonstrated that gut microbiota is only the first layer of a multilayer barrier separating our organism from the content of intestinal lumen and, thus, from the external environment: the so-called “gut barrier”. This barrier is composed, beyond microbiota, by the mucus layer on the epithelial cells, the epithelial cells themselves, the immune cells harboring in the submucosa and by the bidirectional interactions between all these layers (Figure 1). Its integrity is essential to maintain the homeostasis, and its disruption has been associated with many gastrointestinal and extragastrointestinal diseases. Whereas the role of gut barrier disruption appears clear in gastrointestinal disorders, its role in extragastrointestinal diseases could be harder to understand. The basis of this role should be searched in the complex function of immune stimulation/tolerance that gut microbiota exerts.

Hematopoietic stem cell transplantation (HSCT) is a potentially curative therapy for many diseases, mostly hematological, otherwise associated with a poor prognosis. Unfortunately, the widespread use of this treatment is often restricted by the development of graft-versus-host disease (GVHD) a condition in which immunocompetent donor T cells attack host tissues in immunocompromised patients, constituting one of the leading causes of non-relapse mortality.^2^ GVHD depends on several factors, such as age, conditioning regimen, hematopoietic graft source and prophylaxis. The traditional classification of GVHD is based on the timing of onset: acute (aGVHD), within the first 100 days after HSCT, and chronic (cGVHD), after the first 100 days. However, beyond the temporal criterion, aGVHD and cGVHD are different diseases, with characteristic clinical presentation, diagnostic criteria, and tissue pathology. Systemic inflammation and tissue disruption predominate in aGVHD, whereas the immune dysregulation leading to several infections is the prevalent presentation in cGVHD.^3^ Thus, the characteristic clinical manifestations became the diagnostic features instead of the time of the onset, based on National Institutes of Health (NIH) consensus criteria.^4^

In particular, in this review we discuss the role of gut microbiota in the GVHD, focusing on data on humans.

## The Healthy Gut Microbiota

In the last years, the increasing interest on human gut microbiota led to large-scale attempts to characterize it. The association of traditional cultural techniques with new molecular techniques based on the analysis of the small subunit ribosomal RNA (SSU rRNA) gene sequences as phylogenetic markers made bacteria the most known components of gut microbiota, identifying more than 1000 species. Bacteria together with Archaea and Eukaryota constitute the three kingdoms in which life is classified. Bacteria are subclassified in many phyla (plural of phylum, major taxonomic division that contains one or more classes, Box 1), but only a few phyla are mostly represented, accounting for more than 160 species, and, among them, Firmicutes (consisting mainly of Gram-positive clostridia) and Bacteroidetes (consisting mainly of Gram-negative bacteria) are prevalent.^1^,^5^ These two phyla, together with the less represented Actinobacteria and Proteobacteria are not only the most abundant, but also include the most diverse microorganisms in the adult gastrointestinal tract. Other represented phyla are Verrucomicrobia, Lentisphaerae, Synergistetes, Planctomycetes, Tenericutes and the Deinococcus-Thermus group, Melainabacteria, and Gemmatimonacete. Regarding the other two kingdoms, the Euryarchaeota, including the highly represented methanogens, are the most represented Archaea, whereas, among the Eukarya, some Candida spp are the most prevalent.

Box 1 Example of taxonomy of *Escherichia coli.*

**Table:** 

**Cellular organisms classification**	Example
**Kingdom**	Bacteria
**Phylum**	Proteobacteria
**Class**	Gammaproteobacteria
**Order**	*Enterobacteriales*
**Family**	*Enteriobacteriaceae*
**Genus**	*Escherichia*
**Species**	*Escherichia coli*

The earliest years of life are essential for the development of individual microbiota that depends on several factors, such as maternal and family members microbiota, kind of delivery, breastfeeding and early exposure to antibiotics. After this phase, individual microbiota composition is stable in the adult life for decades, and it may be the same also for the entire lifetime unless perturbing factors occur, such as antibiotic therapies or infections.^6^

## The Role of Gut Microbiota in the Immune Regulation

The correct development of gut microbiota is strictly related to the healthy maturation of the immune system, and both develop in the first 2 years of life. In fact gut microbiota constitutes a stimulus that drives the development of the immune system in its capacity to react to pathogens and in the induction and maintenance of the tolerance process. On the other side, immune dysregulation can induce an alteration in gut microbiota.^7^,^8^ The importance of this bidirectional relationship has been highlighted by data from germ-free (GF) animals that showed reduced development of both innate and adaptive immunity with increased susceptibility to microbial infections.^9^,^10^

The integrity of the gut barrier is the basis of the healthy stimulation of the immune system by microbiota.

In fact, the continuous stimulation by luminal commensal antigens should be regulated to avoid the over-stimulation of the immune system. This is warranted by the presence of a physical barrier between gut microbiota and host immune cells, composed of epithelial cells and the mucus layer above them. In particular, the mucus layer consists of an inner and an outer layer, but whereas the outer one is colonized by large numbers of bacteria, the inner one, thicker than the outer one, constitutes a barrier for them.^11^,^12^ Furthermore, even innate lymphoid cells^13^,^14^ and IgAs^15^ contribute to reduce the penetration of microorganisms through the epithelial cells and their presentation to the immune system. Microbiota is essential for the correct development of both innate and adaptive immune response.

Conversely, microbiota needs a healthy immune system to correct its development. In fact, for example, the deficit in IgA response alters the composition of microbiota.^16^–^18^

## Microbiota and the Innate Immune Response

Gut microbiota could regulate lamina propria phagocytes and, in particular, it could increase the production of pro-IL1β in resident macrophages^19^ and neutrophils,^20^ that could be rapidly activated in IL1β in response to pathogens. Microbiota could also influence systemic neutrophils response enhancing their bactericidal activity triggering the NOD1 signaling through peptidoglycan stimulation.

## Microbiota and the Adaptive Immune Response

Data from germ-free (GF) animals demonstrated that when the microbiota is absent, there is a shift through a T-helper (Th)2 response, due to a reduced number of Th1 and Th17 cells, which could be reversible in case of colonization of the gut by flora. In particular, in the small intestine Th17 cells could be stimulated mainly by *segmented filamentous bacteria* (SFB), species belonging to commensal Clostridia-related bacteria,^21^–^24^ and *Lactobacillus johnsonii*.^25^

Beyond T cells, also B cells and immunoglobulins production are influenced by microbiota. In fact, the intestinal mucosa is essential to the correct development of B cells as well as fetal liver and bone marrow, and microbiota is able to regulate intestine-specific B-cell receptor.^26^,^27^ In fact, the presence of commensal microorganisms in the gut stimulates gut-associated lymphoid tissues (GALTs), such as both Peyer’s patches and isolated lymphoid follicles.^28^–^30^ The continuous stimulation induces germinal center formation in isolated lymphoid follicles and Peyer’s patches and IgA production, differently from systemic lymphoid organs where germinal center formation does not occur under physiological condition, but only after a specific- i.e. infectious- stimulation.^31^ In fact, microbial products are required to stimulate the germinal centers in lymphoid follicles and IgA production, in particular through Nucleotide-binding oligomerization domain-containing protein (NOD)1-mediated signaling.^18^,^32^,^33^

## Tolerance Education by Microbiota

Colonic FoxP3^+^ T regulatory (Treg) cells are strongly influenced by the presence of gut microbiota. In fact, they are reduced in colonic lamina propria in the absence of gut microbiota stimulation, whereas the presence of gut microbiota is less relevant for Treg of the small intestine or mesenteric lymph nodes.^34^,^35^ In particular, murine data demonstrated that *Clostridia* and *Bacteroides fragilis* could be the most powerful inducers of Treg,^34^–^39^ probably working through different mechanisms which could be dependent and independent from toll-like receptors (TLRs) signaling. Among TLRs-independent pathways, short-chain fatty acids (SCFAs) - bacterial metabolites deriving from carbohydrates fermentation, including acetate, propionate, isobutyrate and butyrate-seem to be able to increase the acetylation of the Foxp3 locus, increasing the number of Treg directly or, indirectly, increasing the production of TGFβ in the intestinal epithelium.^36^,^40^–^42^ Furthermore, SCFAs induced the expression of the receptor GPR15, responsible for recruitment of Treg in the large intestine.^40^–^50^ Similarly, the folic acid produced by colonic microorganisms could increase the survival of Treg cells.^51^ Furthermore, gut microbiota could stimulate the production of the anti-inflammatory cytokine IL10 by intestinal macrophages.^52^

## The Allogenic Transplant and the Graft-versus-Host Disease

Every year, more than 39000^53^ HSCT are performed only in Europe for an ever expanding number of neoplastic and non-neoplastic diseases, in particular for hematological conditions such as leukemias and lymphomas.^54^

Nevertheless, HSCT is still limited by the development of GVHD, a condition that results from the interaction between the host cells which are targeted by the transplanted donor immune cells, primarily T cells.^55^

GVHD was historically classified in acute and chronic, respectively, if the onset of symptoms was before or after 100 days. However recent advantages questioned these definitions, and current consensus states that clinical features define GVHD as acute or chronic.^4^ aGVHD^56^ occurs mainly in the skin, GI, and liver. GI manifestations of aGVHD include secretory diarrhea, vomiting, abdominal pain and, in severe cases, bleeding. The severity of aGVHD is classified in four grades on the basis of the involvement of the organs mentioned above.^57^ On the other hand, cGVHD manifestations are typically variable, and many organs can be involved, frequently with autoimmune-like diseases characteristics.^58^

## GVHD Pathogenesis and the Role of Gut Microbiota

The mechanisms leading to GVHD are usually divided into steps: organ damage, priming of the immune response, activation of T cells and destruction of target organs by mean of the activated immune cells^2^,^57^,^59^ (Figure 1). The incidence of GVHD is positively correlated with the degree of human leucocyte antigen (HLA) mismatch as the histocompatibility antigens are the main proteins recognized by donor immune cells.^60^ The connection between GVHD and microbiota was firstly suggested in pioneering studies in mice.^61^,^62^ However, studies in humans are still scant and characterized by small sample sizes. These studies mainly investigated variations in the gastrointestinal microbiota before and after HSCT and the impact of its composition on the transplant outcomes (Table 1).

Taur et al. demonstrated that there is a marked reduction after HSCT in the microbiota diversity which leads to the selection of a limited number or, even, of a single “dominating” bacterial genus. Interestingly, patients who developed intestinal domination showed an increased risk of bacteremia which was frequently caused by the same identified “dominating” bacteria.^63^ The authors also described the effects of different antibiotics on the development of specific bacterial prevalences: for example, fluoroquinolones reduced the risk of gram-negative bacteremia by decreasing proteobacterial domination.^63^

Other studies investigated variations of the microbiota in relation to the development of GVHD, the second most common cause of mortality in the context of allogeneic HSCT.^64^ In particular, the onset of GVHD seems to be associated with a progressive reduction of the microbiota diversity with a relative increase in Lactobacillales and a relative decrease in Clostridiales.^65^ Noteworthy, these findings are consistent with those in mice, suggesting that animal studies may, at least, guide the research in humans.

Given these data and considering the already mentioned dramatic impact of GVHD on survival, it is not surprising that microbiota diversity was also found to be an independent risk factor for mortality in patients undergoing HSCT.^66^

Consequent studies focused on the analysis of bacterial composition, researching if specific genera or species could be more implicated than others in the development of GVHD. For example, analysis of bacterial genera found that the abundance of a specific genus, namely *Blautia* (which belong to the *Clostridia* class), is associated with GVHD-related mortality.^67^ Although it was not possible to demonstrate causality in this study, these data may represent a starting point for the development of a GVHD mortality biomarker in the near future.

Similarly, other authors reported that there is an increase after HSCT in the relative abundance of enterococci that was persistent and more pronounced in adults patients with active GVHD.^68^ Similar results have been obtained in children by Biagi et al., who analyzed fecal samples collected from 10 children before HSCT and three times in the following 100 days. After HSCT, a profound alteration of the gut ecosystem occurred in all children, with the loss of about 30% in average of alpha diversity -a measure of diversity within a population in terms of number and distribution- compared to pre-HSCT samples. However, the last samples collected showed a minor degree of difference compared to pre-HSCT specimens, suggesting a natural trend to recover after the disturbance caused by the HSCT. The fecal amount of short-chain fatty acids (SCFA) followed the variations of microbiota: it decreased by 76% after HSCT, being propionate the most reduced (mean loss 86%), and trend to recover distancing the HSCT. Although these differences are common in patients with and without aGVHD, the 5 children who developed aGVHD also showed an overgrowth of *Enterococcus* and *Clostridiales* and a corresponding decrease of *Faecalibacterium* and *Ruminococcus.* At phylum level, patients with aGVHD showed a drop in *Firmicutes* abundance after HSCT but, distancing the HSCT, they showed higher abundance than the initial one, whereas they demonstrated a lower abundance of *Bacteroidetes* compared to non-aGVHD patients. Even if alterations of gut microbiota induced by conditioning regimen and HSCT seem to be crucial to the pathogenesis of GVHD, the pre-HSCT characteristics of gut microbiota could also play a major role. In fact, children who developed aGVHD showed lower diversity and richness before HSCT compared to the other patients and, in particular, they demonstrated a lower abundance of *Bacteroides* and *Parabacteroides*, whose abundance positively correlated with the concentration of propionate and SCFA.^56^

Our group identified that the conditioning regimen, starting from the same baseline microbiota composition, promotes changes in the microbiome, which are different between Autologous (auto-) and Allogeneic Stem Cell Transplantation (allo-SCT). After auto-SCT we documented an increase of *Proteobacteria* (*Klebsiella, Proteus, Acinetobacter, Haemophilus, Pseudomonas, Enterobacteriaceae*) and a reduction of *Bacteroidetes* (*Bacteroides, Saprospirae, Prevotella*). After allo-SCT, instead, there was an increase of *Bacteroidetes* and a reduction of *Firmicutes* (*Bacilli, Lactobacilli, Clostridium, Enterococci, Streptococci*). Moreover, patients who developed GVHD harbored more *Firmicutes and Proteobacteria* and fewer *Bacteroidetes* than patients without this complication. In patients with gut GVHD, *Proteobacteria* were more represented than in patients with liver or skin involvement.^69^

Collectively, these studies showed that the intestinal microbiota is heavily affected by HSCT, being the principal finding, reported in all studies, the reduction in the overall bacterial diversity. At the same time, some studies reported specific alterations which are interestingly correlated with the development of the major complications of HSCT, such as bacteremia and GVHD. While a causative role of the microbiota in these conditions is yet to be demonstrated, these and future studies may give a better comprehension of the complex mechanisms underlying HSCT and GVHD, ultimately allowing better outcomes.

## New Perspectives: the Role of Paneth Cells and Genetic Modifiers of Gut Microbiota

Recently, researchers focused on Paneth cells in an attempt to find a mechanistic relation between microbiota and GVHD. Paneth cells secrete antimicrobial peptides such as alpha-defensins which contribute to the regulation of the GI microbiota. During GVHD, Paneth cells appear to be damaged with a consequent reduction in alpha-defensins production.^70^ Noteworthy, alpha-defensins activity is directed mostly toward non-commensal bacteria, thus decreased levels of these peptides lead to a reduction of commensal bacteria and, intuitively, to an impairment in their beneficial effects.

A subsequent study investigated if Paneth cells number may correlate with the severity, response to treatment and survival of GVHD. Authors found that Paneth cells number was inversely correlated with the clinical severity stage with a strong correlation between the two parameters. Response to treatment at 4 weeks was also found to be positively correlated with Paneth cells number, being highest in patients with a complete response and lowest in patients who did not respond. Finally, a threshold of 4 Paneth cells per high power field (HPF) was found to discriminate between high and low-risk patients regarding non-relapse mortality (NRM), with also a significant difference in the overall survival.^71^

Similarly, Ferrara et al. demonstrated that a specific lectin secreted by Paneth cells, namely regenerating islet-derived 3-alpha (REG3alpha), has diagnostic value in acute GI GVHD permitting to differentiate between GVHD-related diarrhea and other causes of diarrhea.^72^ The authors also demonstrated a prognostic value of REG3alpha in GVHD, in particular, a positive correlation between plasma levels and NRM was found. This result may appear in contrast with the previous data, in particular with the evidence supporting a protective role of Paneth cells. However, the authors hypothesized that the GI mucosal barrier disruption which occurs in GVHD permits to the nearby Paneth cells secretions to enter the bloodstream.^72^

Similarly, there is an increasing interest in the role of the Fucosyltransferase 2 (FUT2) gene, a genetic modifier of the GI microbiota which seems to be associated with different GI diseases.

Various antigens are expressed in the intestinal mucin layer, for example, ABH antigens are oligosaccharides that constitute a site of attachment and a carbon source for intestinal bacteria.^73^ Their expression is regulated by an enzyme which in humans is encoded by the FUT2 gene.^74^ Polymorphisms in the FUT2 gene are correlated with alteration of the GI microbiota both in the compositional and functional level.^75^ Recently, homozygosity for the loss-of-function alleles (non-secretors, A/A genotype) was demonstrated to be associated with increased susceptibility to Crohn’s disease.^76^ Rayes et al. also showed that FUT2 polymorphisms influence the risk of GVHD and bacteremia in the context of HSCT. Specifically, the Authors found that there was a reduced risk of acute GVHD with A/A genotype (non-secretors) and an increased risk with the G/G genotype (secretors) while an increased risk for bacteremia was found with A/A and A/G (secretors) genotypes.^73^

## Gut Microbiota Modulation as a Preventive Strategy Against GVHD

Considering the major role of gut microbiota in the pathogenesis of GVHD, its modulation with prebiotic, probiotic and antibiotic could be a strategy to reduce the incidence of GVHD.

Some studies reported reduced numbers and less severe GVHD after the use of broad-spectrum antibiotics to “decontaminate” the gastrointestinal tract.^77^,^78^ However, GI decontamination fell into disuse in the 1990s mostly because of heterogeneous successful decontamination rates, high costs and lack of corroborating data of its utility.^79^ The reasons behind the failure of total gut decontamination in the clinical setting are still unknown. However, many Authors hypothesized that an explanation may be that this practice affects the microbiota as a whole, without distinction between “good” bacteria and pathogens.^66^,^67^,^80^ In fact, as discussed above, there is evidence that bacteria diversity is the cornerstone of the “healthy” microbiota while a decreased diversity is often found in many GI and extra gastrointestinal diseases.^81^

Probiotics drew growing interest in the world of gut microbiota modulation and suggested in murine models that they could be effective in decreasing the aGVHD severity.^65^,^82^ However, the use of probiotics in immunosuppressed patients is limited by possible safety issues. Ladas et al. evaluated the safety of the administration of *Lactobacillus plantarum* during the conditioning regimen and the post-HSCT neutropenic period in 30 children and adolescents. The majority of patients (70%) did not develop GVHD. Three patients died within 100 days from the HSCT, but the causes of death are judged unrelated to probiotics. Furthermore, no episodes of *Lactobacillus plantarum* bacteremia were observed. Even if these results are preliminary, they laid the foundation for larger clinical trials to evaluate the efficacy and the safety of probiotics in prevention and treatment of GVHD.

Prebiotics are defined as nondigestible food components that are able to modulate the intestinal microbiota with a possible beneficial effect on the human body.^83^ In particular, oligosaccharides represent an important fraction of human milk and are known to exert a prebiotic effect.^84^ Modulation of the microbiota is also achieved indirectly, as oligosaccharides can prevent adhesion of enteropathogens acting as soluble decoys.^85^ Both these mechanisms indirectly reduce inflammation,^86^ even if recent evidence suggest that oligosaccharides also have a direct inhibitory effect on inflammation.^87^–^89^

Fecal microbiota transplantation (FMT), the infusion of feces from a healthy donor into the gut of a recipient patient, was recently proven to be safe and effective in *Clostridium difficile* infection after HSCT when conventional therapy failed.^90^,^91^ In fact, patients undergoing HSCT are exposed, due to antibiotic prophylaxis and to the procedure itself, to colonization by multi-drug resistant bacteria with a negative impact on the main transplant outcomes, such as overall survival and non-relapse mortality, but also on the development of clinically relevant aGVHD.^92^

Interestingly the same authors found, in a preliminary study, that FMT was able to eradicate resistant bacteria harbored in the gut of an immunocompromised patient affected by multiple myeloma.^93^

While further studies are awaited, these data suggested that FMT may represent, in the near future, a novel strategy to modulate the gut microbiota with a possible impact on GVHD.

## Conclusions

Gut microbiota and its continuous stimulation of immune system is essential for the development and the “maintenance” of a healthy gut. HSCT-related procedures could alter this balance laying the foundation for the development of GVHD. The possibility of modulation of gut microbiota as a preventive or curative strategy for GVHD is intriguing and should be developed in the future, to reduce the morbidity and mortality of this condition.

## Figures and Tables

**Table 1:** Summary of human studies assessing gastrointestinal microbiota in Graft versus Host Disease.

Author and Year	Transplant	Most common indication	Specimens type	Specimens analysis	Population	Aim	Groups	Patients	Results	Ref
Jenq, 2012	allo HSCT	Leukemia	fecal	16s rRNA gene sequencing	Adults	microbiota variation	GVHD (8 pts) vs No GVHD (10 pts)	18	GVHD is associated with reduced flora diversity (increases in Lactobacillales and decreases in Clostridiales).	[65]
Vossen, 2014	allo HSCT	Leukemia	-	-	Children	occurrence of GVHD	GID (57 pts) vs No GID (55 pts)	112	Successful total GID resulted in significantly less acute GVHD (p = 0.013; log-rank test).	[78]
Holler, 2014	allo HSCT	Leukemia	fecal	16s rRNA gene sequencing	Adults	microbiota variation	pre and post transplant comparison	31	Increase in Enterococci and decrease in other Firmicutes and phyla after allo HSCT. Shift most pronounced in active GVHD.	[68]
Jenq, 2015	allo HSCT	Leukemia	fecal	16s rRNA gene sequencing	Adults	microbiota variation outcome	pre and post and transplant comparison	115	Intestinal flora diversity and Blautia abundance is associated with reduced GVHD lethality.	[67]
Taur, 2014	allo HSCT	Leukemia	fecal	16s rRNA gene sequencing	Adults	microbiota variation outcome	pre and post and transplant comparison	80	Intestinal microbiota diversity is an independent predictor of mortality.	[66]
Taur, 2012	allo HSCT	Leukemia	fecal	16s rRNA gene sequencing	Adults	microbiota variation outcome	pre and post and transplant comparison	94	Bacterial “domination” is associated with increased risk of bacteremia.	[63]
Chiusolo, 2015	allo/auto HSCT	Leukemia	fecal	16s rRNA gene sequencing	Adults	Microbiota variation outcome	pre and post and transplant comparison	8	Increase of Proteobacteria and reduction of Bacteroidetes after auto HSCT. Increase of Bacteroidetes and reduction of Firmicutes after allo HSCT. GVHD associated with more Firmicutes and Proteobacteria and less Bacteroidetes.	[69]

Abbreviations: allo HSCT, allogenic hematopoietic stem cell transplantation; auto HSCT, autologous hematopoietic stem cell transplantation; GVHD, graft versus host disease; GID, gastrointestinal decontamination.
